# Quantifying the duration and variability of thoracic organ procurement cases

**DOI:** 10.1016/j.xjon.2026.101866

**Published:** 2026-05-15

**Authors:** Pablo G. Sanchez, Carson Herman, Kalyna Sconzert, Pratik Patel, Alejandro Bribriesco

**Affiliations:** a Biological Sciences Division, The University of Chicago, Chicago, Ill; b Paragonix Technologies, Inc, Waltham, Mass

**Keywords:** heart transplant, lung transplant, medical economics, thoracic organ procurement

## Abstract

**Objective:**

To quantify and analyze key temporal intervals and variability in heart and lung thoracic organ procurement using multicenter registry data and assessing logistical complexity across operational phases and preservation modalities to inform strategies for optimizing transplant efficiency.

**Methods:**

This retrospective study analyzed timestamp data from 25,935 events across 7665 thoracic organ procurement cases (4992 heart, 2673 lung) performed between 2021 and 2025 using the Paragonix Mobile Application. Four temporal intervals were examined: total time away from transplant center, time at donor location, time to organ acceptance, and organ explant duration. Cases were stratified by organ type (heart and lung) and donor type (donation after brain death and donation after circulatory death).

**Results:**

Median total time away from transplant center was 7.4 hours for heart and 8.5 hours for lung procurement. Time at donor location showed was similar for each organ type (heart 3.2 hours vs lung 3.4 hours). Acceptance time demonstrated the shortest medians (heart 0.5 hours vs lung 0.8 hours). Organ explant duration was consistent (heart 2.0 hours vs lung 2.1 hours). All intervals exhibited right-skewed distributions with outliers. Donation after circulatory death cases demonstrated shorter surgical intervals compared with donation after brain death cases.

**Conclusions:**

This analysis provides empirical benchmarks for thoracic organ procurement timelines, revealing substantial real-world variability while demonstrating comparable durations between heart and lung procurement. These data may inform resource-allocation strategies, suggest a role for third-party procurement services, and optimize workflows for expanding donation after circulatory death programs.

**Figure:**
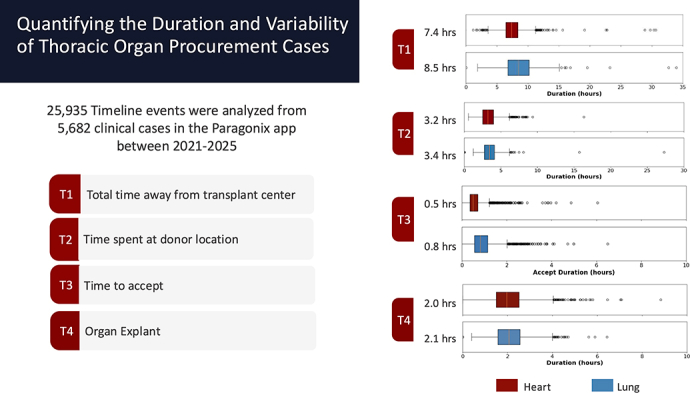
Benchmarking timeline segments in thoracic procurement highlights process inefficiencies.

**Central Message**

Timestamp-based benchmarking reveals key opportunities in thoracic organ procurement for improving efficiency, reducing costs, and guiding policy in the era of expanded geographic and model innovation.

**Perspective**

This multicenter, real-world benchmarking of procurement intervals demonstrates substantial variability, highlights actionable targets for efficiency gains, and sets the stage for process-driven cost optimization and policy advances as the transplant field embraces regionalization, broader sharing, and specialized care models.

Thoracic organ transplantation, encompassing both heart and lung transplantation, represents the definitive therapeutic intervention for patients with end-stage cardiac and pulmonary failure.^1^^,^^2^ The success of these life-saving procedures depends not only on surgical expertise and immunological compatibility but also on the efficient recovery of donor organs within a relatively short window of viability.^3^, ^4^, ^5^ Although substantial progress has been achieved in refining surgical techniques, donor selection criteria, and posttransplant care, the operational framework supporting organ procurement has received comparatively limited evaluation.^6^ Thus, a comprehensive characterization of the temporal dynamics and variability in the donor organ–recovery processes may identify opportunities to optimize organ use, reduce ischemic exposure, and ultimately enhance transplant outcomes.^7^, ^8^, ^9^

The time interval between donor crossclamp and recipient implantation is determined by a complex set of clinical, logistical, and technological factors that are continually evolving.^10^ In recent years, the use of third-party procurement strategies to increase organ acceptance and recovery rates has become widespread, with 82.6% of US transplant centers indicating that they have used outside or contracted services for a portion of their cases, and clinical studies showing that 1-year graft survival rates are not compromised by third-party procurement.^11^ The shift toward third-party procurement comes with several potential advantages, including the recovery of more distant organs and better accommodation of fluctuations in organ availability. In lung transplantation, the transition from the lung allocation score (LAS) to the composite allocation score (CAS) has been associated with broader geographic sharing and increased procurement distances, which may impact procedural timelines.^12^ As procurement protocols evolve, so does organ-preservation technology.^11^ Advanced preservation devices allow not only for more stable control of temperature but also for continual logistical tracking that can provide real-time data to transplant surgeons and their teams. At the same time, The United Network for Organ Sharing has publicly urged that organ-tracking systems should be implemented to prevent donor organs from being lost or delayed in transit.^13^

To date, no study has leveraged real-time temporal data from advanced organ-preservation technologies to characterize procurement timelines in detail. This gap in knowledge can make it difficult for centers to optimize transplant logistics or to understand exactly at which points time is lost in delayed cases. Such insights are particularly relevant when considering the use of organs from donation after circulatory death (DCD) or other marginal donor groups, in which extended preservation times can increase the risk of allograft dysfunction or mortality. DCD donors have a high nonuse rate, particularly for thoracic organs.^14^ Thus, a more detailed understanding of procurement timelines as organ recovery processes continue to evolve may allow for a more effective use of DCD thoracic organs to sustainably meet a growing demand.

The present study aims to quantify the duration and variability of discrete temporal intervals during thoracic organ-procurement procedures using multicenter registry data from heart and lung transplant recipients. By systematically analyzing distinct operational phases, including total time away from the transplant center, duration at the donor location, organ explant time, and acceptance time, this investigation seeks to characterize the logistical complexity inherent in thoracic allograft procurement. Furthermore, this work examines temporal patterns that may inform future strategies for optimizing procurement operations, evaluating the potential contributions of specialized support services, and advancing data-driven approaches to enhance efficiency and outcomes in solid-organ transplantation.

## Methods

### Study Design and Data Source

This retrospective study analyzed temporal characteristics of thoracic organ-procurement procedures using timestamp data collected through the Paragonix Mobile Application. The application captures real-time procedural events during clinical transplant cases, providing granular temporal data across the procurement process. Data encompassed heart and lung transplantation cases performed between 2021 and 2025. Institutional review board approval was not required, as all data were deidentified and collected for quality improvement as per institutional policy. Informed consent also was not required, as all timestamp data were deidentified and inclusion in this analysis was approved per institutional quality improvement protocols.

### Study Population

Data from 25,935 timeline events were pulled from 5682 clinical app cases between 2021 and 2025. Moreover, the counts from donation after brain death (DBD) and DCD donors were analyzed, which included 4727 DBD and 265 DCD heart cases and 2274 DBD and 399 DCD lung cases. The cases were eligible for inclusion if valid timestamp data were available for at least 2 of the predefined temporal categories required to calculate procedural durations. Data entries with incomplete timestamps were excluded from analysis. Surgical procurement techniques and preservation methods were left to the discretion of the recipient center and were not captured in this analysis.

### Temporal Interval Definitions

Four distinct temporal segments were defined using timestamped events captured by the mobile application:

T1 (total time away from transplant center): Calculated as the interval from departure from the transplant center to return to the transplant center (TDepartTxC to TArriveTxC). This metric represents the complete procurement mission duration.

T2 (time spent at donor location): Calculated as the interval from arrival at the donor center to departure from the donor center (TArriveDonorCenter to TDepartDonorCenter). This interval encompasses donor evaluation, surgical preparation, procurement, and organ-packaging activities.

T3 (time to accept organ): Calculated as the interval from surgical incision to organ acceptance or decline decision (TIncision to TAccept or TIncision to TDecline). This metric reflects the organ evaluation and decision-making timeline.

T4 (procedural duration): Calculated as the interval from surgical incision to organ removal (TIncision to TOrganRemoved). This metric captures the procurement procedure, including procedural pauses for coordination, delays, or other inactive periods.

### Data Processing and Statistical Analysis

Timestamp data were extracted in comma-separated value format and analyzed using Python programming language. All temporal values were normalized to hours to enable standardized comparison across cases. Descriptive statistics were used to characterize each temporal segment. Mean and standard deviation values were calculated for each interval and stratified by organ type (heart vs lung) and donor type (DBD vs DCD). Box plot visualizations were generated to illustrate the distribution, central tendency, and variability of each temporal interval across study cohorts.

A subanalysis was conducted within the lung cohort to quantify these durations before and after the transition from the LAS to the CAS. March 9, 2023, was used as the cutoff date; cases before this date were classified as pre-CAS and those on or after this date as post-CAS. Durations were summarized using the median and standard deviation (SD), calculated using the same methods described previously.

## Results

### Temporal Characteristics of Thoracic Organ Procurement

**Figure 1:**
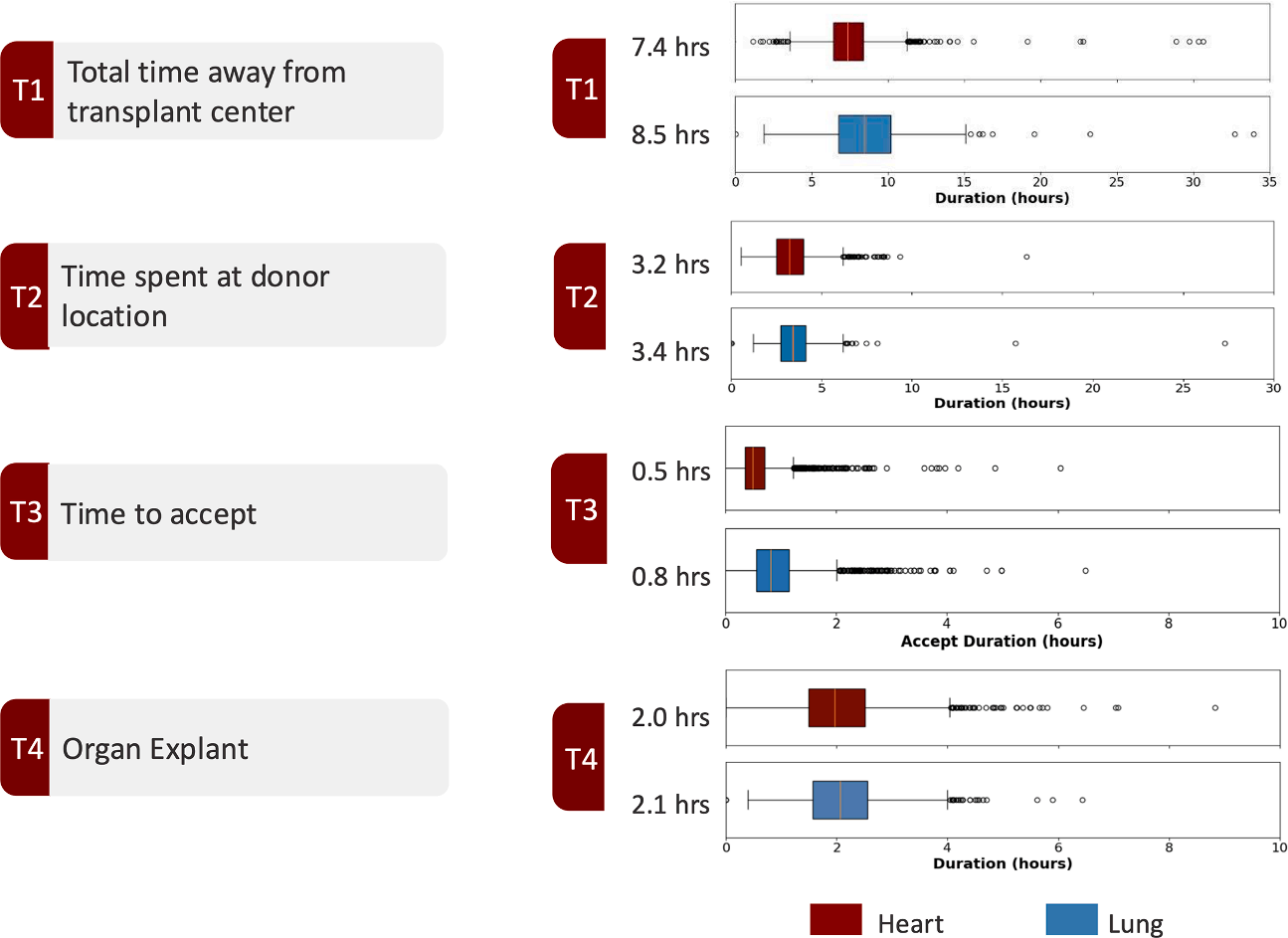
Temporal characteristics of thoracic organ procurement by organ type. Box plots show the distribution of 4 key time intervals (T1: total time away from transplant center, T2: time spent at donor location, T3: time to accept organ, T4: organ explant duration) for heart and lung procurement cases.

This study quantified the duration and variability of key time intervals during thoracic organ-procurement procedures in a cohort of recipients who received heart and lung transplants. Four distinct time intervals were examined to characterize the procurement process (Figure 1).

The median total time away from the transplant center was 7.4 hours for heart procurement and 8.5 hours for lung procurement. This metric represents the entire procurement mission duration from departure to return to the transplant center. Both organ types exhibited variability, with durations extending to approximately 30 hours in heart cases and 35 hours in lung cases. For heart procurement, the median time spent at the donor location was 3.2 hours, and for lung procurement, the median was 3.4 hours. Both cohorts demonstrated outliers extending to approximately 10 hours.

The interval from surgical incision to organ acceptance demonstrated the shortest median durations among all intervals analyzed. Heart procurement showed a median acceptance time of 0.5 hours, and lung procurement exhibited a median of 0.8 hours. The procurement procedure duration was consistent between organ types. Heart procurement exhibited a median explant time of 2.0 hours, and lung procurement showed a median of 2.1 hours.

### Variability and Distribution Patterns

Box plot analysis revealed that all 4 temporal intervals demonstrated right-skewed distributions with upper outliers in both heart and lung procurement cohorts. The acceptance duration (T3) exhibited the most pronounced variability relative to its median value. In contrast, the organ explant duration (T4) demonstrated the most consistent performance, with fewer extreme outliers. The total time away from the transplant center (T1) and time spent at donor location (T2) showed intermediate levels of variability.

### Temporal Characteristics by Donor Type

**Figure 2:**
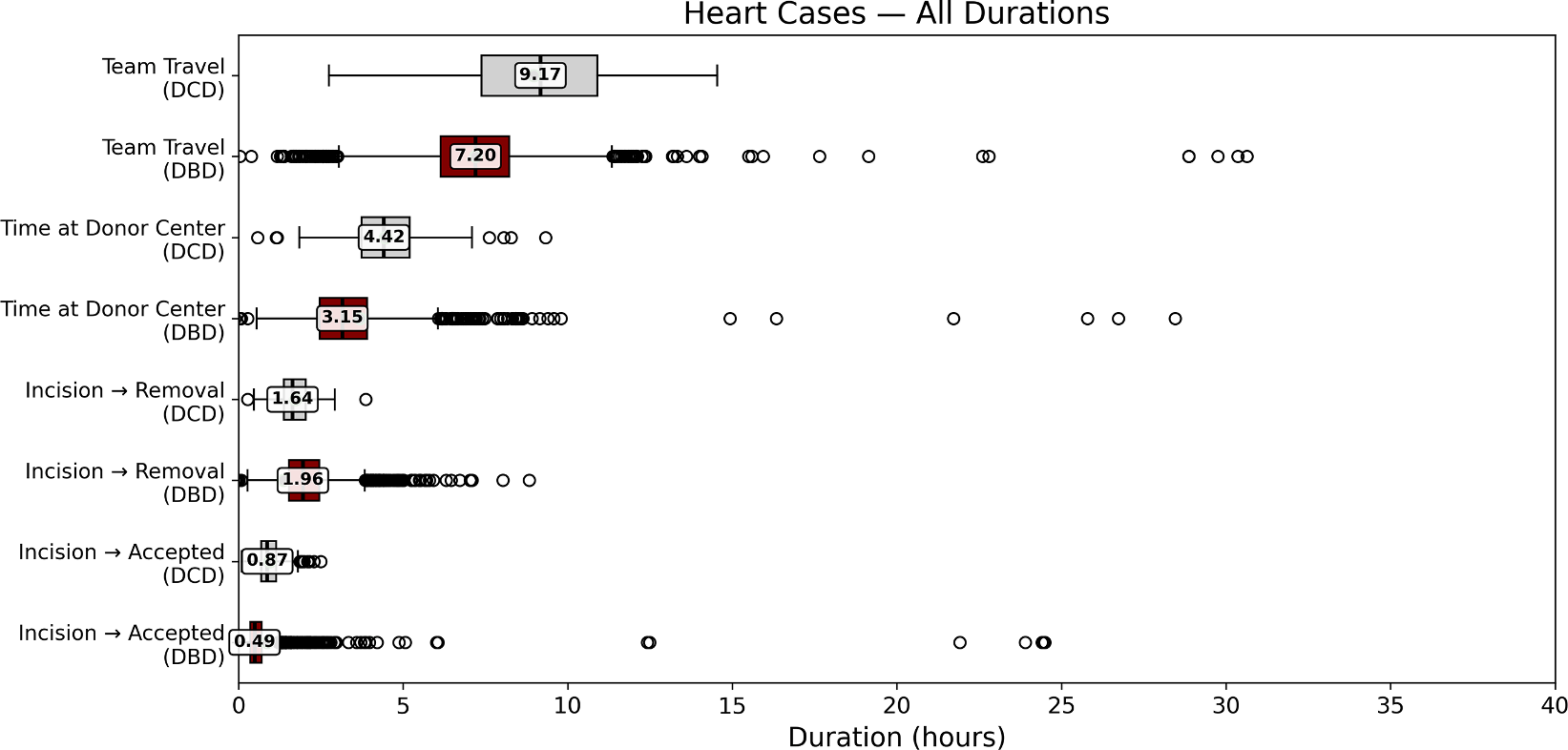
Temporal characteristics of heart stratified by donor type. Box plots show the distribution of team travel time, time at donor center, incision to removal interval, and incision to accepted interval for donation after circulatory death (*DCD*) and donation after brain death (*DBD*) donors. The median for each duration is highlighted in the *white box*.

**Figure 3:**
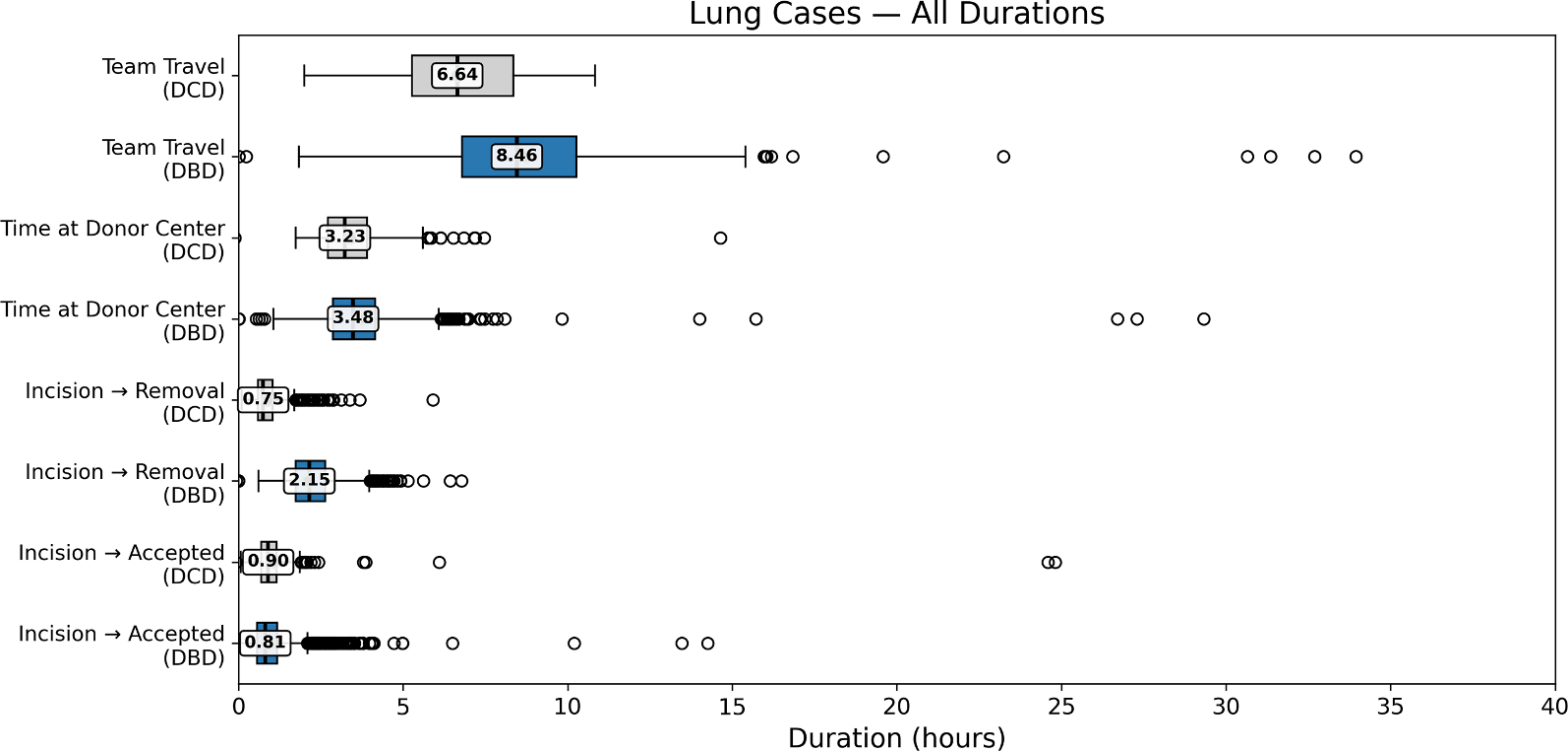
Temporal characteristics of lung procurement stratified by donor type. Box plots show the distribution of team travel time, time at donor center, incision to removal interval, and incision to accepted interval for donation after circulatory death (*DCD*) and donation after brain death (*DBD*) donors. The median for each duration is highlighted in the *white box*.

The analysis further stratified procurement intervals by donor type, comparing cases involving DCD with those involving DBD. This stratification revealed distinct temporal patterns across 4 key operational intervals: team travel time, time at donor center, incision to accepted interval, and incision to removal interval (Figures 2 and 3).

Heart procurement demonstrated modest differences in team travel time between donor types, with DCD cases showing a median duration of approximately 9.2 hours compared with 7.2 hours for DBD donors. Lung procurement exhibited an inverse pattern, with DBD cases requiring longer travel times (median 8.5 hours) compared with DCD cases (median 6.6 hours).

Heart procurement revealed pronounced differences by donor type, with DCD cases requiring a median of approximately 4.4 hours at the donor facility compared with 3.2 hours for DBD cases, representing a 33% increase in on-site duration. Lung procurement demonstrated a contrasting pattern, with DBD cases showing longer donor center durations (median 3.5 hours) compared with DCD cases (median 3.2 hours).

### Incision to Removal Interval

The time needed to surgically remove the donor organ was consistent across both organ types and donor types. Heart procurement showed a median incision-to-removal time of approximately 1.6 hours for DCD cases and 2.0 for DBD cases, with tight clustering around the median values. Lung procurement exhibited similar standardization, with DCD cases showing a median of approximately 0.8 hour and DBD cases approximately 2.2 hours.

### Incision to Acceptance Interval

Heart procurement demonstrated almost a 2-fold difference in acceptance timing, with DCD hearts showing a median acceptance time of approximately 0.9 hours compared with 0.5 hours for DBD hearts. This extended evaluation period for DCD hearts reflects the additional assessment required to evaluate organ viability and function before committing to transplantation. Lung procurement exhibited a different pattern, with DCD lungs demonstrating almost equivalent acceptance (approximately, median 0.9 hours) as DBD lungs (approximately, median 0.8 hours).

### Temporal Characteristics Pre- and Post-Lung Allocation Policy Change

The analysis further stratified procurement intervals within the lung cohort by allocation era, comparing cases performed before and after the transition from the LAS to the CAS. Across the evaluated operational intervals, including time at the donor center, incision to acceptance, and incision to removal, durations were similar between eras. Team travel time differed between groups. Pre-CAS cases had a median team travel duration of 7.5 hours (SD 4.6 hours), whereas post-CAS cases had a median duration of 8.5 hours (SD 4.4 hours).

## Discussion

This study provides a comprehensive characterization of temporal intervals in thoracic organ procurement, demonstrating overall consistency across key procedural phases with measurable variability in select intervals. Team travel time represented the longest and most variable component of the procurement process, whereas incision-to-removal intervals were relatively consistent, suggesting standardized surgical practices. Differences between DCD and DBD procurement highlight distinct operational considerations, particularly with respect to procedural timing and resource use. In addition, after the transition from LAS to CAS, most procedural intervals remained stable, whereas team travel time increased within the lung cohort.

Thoracic organ procurement has traditionally involved transplant center–based retrieval teams traveling to donor hospitals to perform organ recovery, an approach termed same-team transplantation.^15^ Geographic distribution of donors and recipients typically requires procurement teams to traverse distances exceeding 200 nautical miles, frequently necessitating private aviation services.^15^ These logistical realities underscore the substantial operational and financial burden inherent in current organ-retrieval practices. This highlights the potential opportunities for alternative procurement models to optimize efficiency and reduce temporal and resource constraints.^11^ In a comprehensive analysis of 159 consecutive lung transplantation cases performed over a 2.5-year period, Shiraishi and colleagues^16^ demonstrated equivalent 1-year posttransplant survival outcomes between recipients of donor lungs procured by external surgical teams and those procured by the recipient center's transplant team.

Recently, it was reported that third-party procurement in lung transplantation yields comparable early transplant outcomes. In a single-center analysis of 113 lung procurement cases, hospital length of stay and 90-day survival rates were similar across all procurement groups. The study demonstrated that delegating procurement to locoregional teams represents a viable strategy for high-volume transplant programs to expand organ access and minimize strain on institutional surgical teams, particularly when evaluating DCD donors or marginal organs with lower acceptance probability.^17^

The temporal data presented in this study hold particular significance for the expansion of DCD thoracic organ transplantation programs. These findings demonstrate that, among organs ultimately transplanted, DCD procurement cases exhibit shorter and more standardized surgical intervals compared with DBD cases, with median incision-to-removal times of approximately 0.5 hours for DCD versus 2.0 hours for DBD donors. This temporal efficiency reflects the rapid procurement protocols necessitated by circulatory arrest but also highlights a critical operational challenge: the inherent unpredictability of DCD donor progression. The elevated organ nonuse rate in DCD cases poses substantial resource allocation challenges for transplant programs. When institutional procurement teams travel to evaluate potential DCD donors, uncertainty regarding the timing of circulatory death and ultimate organ suitability creates risk of unsuccessful procurement missions. These data demonstrate that team travel times average 7.4 to 8.5 hours, representing significant opportunity costs in terms of surgeon time, operating room availability, and financial expenditure. The deployment of third-party procurement services or specialized organ recovery teams may represent a potential strategy that allows the recipient center personnel to remain available for procedures while external teams manage the evaluation and procurement process at donor sites.

The use of third-party procurement services may also provide value in the context of evolving allocation policies. The temporal patterns observed after the transition from LAS to CAS highlight additional operational considerations for lung procurement in the CAS era. Although most procedural intervals, including time at the donor center and incision-to-event durations, remained consistent between eras, team travel time increased in the post-CAS period. This finding likely reflects the broader geographic sharing associated with the CAS policy, which has expanded the distance between donor and recipient centers. Despite this increased travel burden, the stability of donor-center and intraoperative intervals suggests that on-site procurement workflows remain largely unaffected by allocation policy changes.

This study has some limitations. The timestamped events analyzed were manually input by clinical staff throughout the transplant timeline and are therefore subject to human error in data entry. In addition, the dataset was limited to cases in which both timestamps required to calculate an event duration were recorded, which may have introduced selection bias if missing data were not randomly distributed. These limitations may affect the generalizability of findings to the broader population of thoracic organ procurement cases. In addition, this study does not capture measures of organ quality, use rates, or posttransplant outcomes, which are important considerations when evaluating procurement strategies. These factors were not directly evaluated and represent important areas for future study, including the value of third-party procurement services, encompassing quality and cost. Also, the educational value of organ procurement in training thoracic transplant surgeons was not assessed, as the analysis was focused on logistical and temporal aspects of procurement.

Although several procedural intervals in thoracic procurement demonstrate overall consistency, variability remains present across all measured durations. Variability in T1 (team travel time) likely reflects differences in geographic distance between donor and recipient centers, as well as travel conditions and logistical complexity. Variability in T2 (time at the donor center) may be attributable to differences in donor management, coordination of multiorgan procurement, and case-specific surgical factors. Similarly, variation in T3 (incision to acceptance) may reflect the need for extended intraoperative assessment in select cases.

Despite this variability, the relatively narrow median ranges observed in T4 (incision to removal) suggest a degree of standardization in surgical procurement practices across thoracic organs. However, the presence of extended outlier durations indicates that certain cases may involve increased complexity, including challenging anatomy, coordination with other procurement teams, or donor-related factors.

This comprehensive analysis is the first to establish empirical benchmarks for thoracic organ procurement temporal characteristics, revealing substantial variability while demonstrating comparable durations between heart and lung procurement. The marked differences between DCD and DBD cases highlight distinct operational considerations for expanding DCD programs. Timestamped clinical data gathered through digital platforms enables precise measurement of procurement durations, supporting optimization of operating room scheduling, development of reimbursement strategies, and quality improvement initiatives. The integration of comprehensive quality management systems with specialized procurement teams creates a framework for enhanced efficiency as the transplant community works to expand the donor pool and optimize thoracic organ use.

## Conflict of Interest Statement

The authors reported no conflicts of interest.

The *Journal* policy requires editors and reviewers to disclose conflicts of interest and to decline handling or reviewing manuscripts for which they may have a conflict of interest. The editors and reviewers of this article have no conflicts of interest.
